# The Effect of Dense and Hollow Aggregates on the Properties of Lightweight Self-Compacting Concrete

**DOI:** 10.3390/ma17184569

**Published:** 2024-09-17

**Authors:** Aleksandr Sergeevich Inozemtcev, Sergey Dmitrievich Epikhin

**Affiliations:** Department of Building Materials Science, National Research Moscow State University of Civil Engineering, Yaroslavskoe Shoose 26, 129337 Moscow, Russia; sergep97@mail.ru

**Keywords:** self-compacting concrete, lightweight concrete, structural lightweight concrete, mobility, rheological properties, strength, homogeneity, hollow microspheres, ratio of quartz powder, ratio of fractionated sand

## Abstract

The development of self-compacting lightweight concretes is associated with solving two conflicting tasks: achieving a structure with both high flowability and homogeneity. This study aimed to identify the technological and rheological characteristics of the flow of concrete mixtures D1400…D1600 based on hollow microspheres in comparison with heavy fine-grained D2200 concrete and to establish their structural and physico-mechanical characteristics. The study of the concrete mixtures was carried out using the slump flow test and the rotational viscometry method. The physical and mechanical properties were studied using standard methods for determining average density and flexural and compressive strength. According to the results of the research conducted, differences in the flow behaviors of concrete mixtures on dense and hollow aggregates were found. Lightweight concretes on hollow microspheres exhibited better mobility than heavy concretes. It was shown that the self-compacting coefficients of the lightweight D1400...D1600 concrete mixtures were comparable with that of the heavy D2200 concrete. The rheological curves described by the Ostwald–de Waele equation showed a dilatant flow behavior of the D1400 concrete mixtures, regardless of the ratio of quartz powder to fractionated sand. For D1500 and D1600, the dilatant flow behavior changed to pseudoplastic, with a ratio of quartz powder to fractional sand of 25/75. The studied compositions of lightweight concrete can be described as homogeneous at any ratio of quartz powder to fractional sand. It was shown that concrete mixtures with a pronounced dilatant flow character had higher resistance to segregation. The value of the ratio of quartz powder to fractional sand had a statistically insignificant effect on the average density of the studied concretes. However, the flexural and compressive strengths varied significantly more in heavy concretes (up to 38%) than in lightweight concretes (up to 18%) when this factor was varied. The specific strength of lightweight and heavy concrete compositions with a ratio of quartz powder to fractional sand of 0/100 had close values in the range of 20.4...22.9 MPa, and increasing the share of quartz powder increased the difference between compositions of different densities.

## 1. Introduction

Modern concrete science has achieved significant results in the study, creation, and application of various types of concrete. Structural heavy concrete is considered common and traditional [1,2]. A significant breakthrough in the development of concrete technology was the creation of self-compacting concretes (SCCs) at the end of the 1980s and the beginning of the 1990s [1,3,4,5,6]. SCC technology allows the filling of formwork spaces without forced compaction while preserving the homogeneity of the structure, which greatly simplifies the concreting process, especially in heavily reinforced structures [1,2,7,8]. The first experience of the mass application of SCC technology in Russia can be considered the continuous concreting of the lower zone of the foundation slab under Tower A of the Federation complex of the Moscow International Business Center, “Moscow-City” [9].

The main feature of SCC mixtures is high mobility; therefore, the focus of research on this material is on studying its rheological and technological characteristics. World research experience allows for establishing formulation factors that contribute to the production and modification of SCC [10,11,12]. A critical review of works dedicated to the rheology of concrete mixtures was presented in [13,14], analyzing studies that have established the influence of individual components of the mixture on its rheological characteristics, such as cement [15], additional binders (fly ash, ground blast furnace slag, and silica dioxide) [16,17], coarse and fine aggregates, and chemical additives (superplasticizer, viscosity modifier, and air-entraining agent). It has been confirmed that the type, chemical composition, content, packing density [18], size [19], surface texture, and granulometric composition of mineral additives [20] have a significant impact on the rheological properties of concrete.

From the mid-20th century to the present day, the idea of transitioning from high-density (heavy) concrete to lower-density (lightweight) concrete to reduce the weight of structures while maintaining the strength characteristics of heavy concrete has remained relevant [21]. The experience with high-strength lightweight concretes and SCCs is currently highly developed. For example, in one study [21], a case was presented of a bridge in southern Norway, the Stolma Bridge, with a cantilever construction type with a main span length of 301 m. The bridge used concrete with a density of 1930 kg/m^3^ and a strength of 70 MPa when concreting the central part of the bridge, distributing loads such that the columns, whose bearing capacity perceives only 90% of the above-water part’s mass, were used. A scientist from the Department of Civil and Environmental Engineering at Hong Kong Polytechnic University [22] presented the latest achievements in the field of high-strength lightweight concrete. The research considered the development of lightweight structural concretes, including the use of high-quality binders and the selection of strong lightweight materials, as well as the addition of fibers. A comparison of the physico-mechanical properties of lightweight concretes with coarse aggregates [23] made from slag, tuff, pumice, and hollow microspheres was conducted [24]. The density of compositions with coarse aggregates varied from 1600 to 2000 kg/m^3^, while the compressive strength exceeded 60 MPa. The use of lightweight microspheres allowed for a reduction in the density of the mixture (from 1200 to 1600 kg/m^3^) while maintaining high compressive strength. Consequently, the structural efficiency of using lightweight microspheres was higher than that of mixtures prepared with coarse lightweight aggregates. In addition to the advantage of weight reduction, high-strength lightweight concrete exhibits superior resistance to water and chloride ion [25] penetration, satisfactory resistance to cyclic freezing and thawing [26], and reduced creep [27] compared with regular concrete of similar strength levels.

The most critical rheological properties describing concrete mixtures—the yield stress (τ_0_), plastic viscosity (µ), and thixotropy—have been identified. Recently, the development of concrete technologies has been directed toward studying structural lightweight self-compacting concretes (LWSCCs), which combine the beneficial properties of both lightweight structural and self-compacting concretes [28]. Such materials promote the rapid construction of lightweight structures with high strength [29].

Russian scientists from the National Research Moscow State University of Civil Engineering conducted research to determine the possibility of producing cement-dispersive systems on lightweight aggregates with increased fluidity [30]. The research subject was lightweight concretes on hollow microspheres. Standard methods for determining the mobility of concrete mixtures, such as the diameter of the slump from a truncated cone, allowed for establishing the capability of the dispersive system to flow under a set regime of influence, i.e., shaking and varying factors: the W/C ratio and plasticizer concentration. It was found that lightweight concrete mixtures on hollow microspheres (average density of 1450 ± 25 kg/m^3^) could flow on their own, which was reflected in achieving similar slump diameters before and after shaking (the difference was 2%). The intensity of the influence of variable factors (the W/C ratio and plasticizer concentration) on free flow was higher than that on flowability under the influence of shaking. However, achieving LWSCC faces the problem of segregation, as reducing the average mix density promotes the flotation of lightweight aggregate.

The problem of LWSCC segregation was considered and solved by adding fiber reinforcement [31,32,33]. This approach maintained the integrity of the low-density concrete mix structure while increasing strength and improving crack resistance. The downside of this technological solution is reduced workability and mixture fillability. Achieving LWSCC involves solving two conflicting tasks: achieving a structure that is both highly fluid and homogeneous while ensuring the capability to fill and compact forms (formwork) independently without external influences.

In designing LWSCC mixtures, special attention is given to the granulometric and volumetric composition of lightweight and fine mineral aggregates. The research results are presented by [34,35,36] on determining the dependence of technological characteristics of concrete mixtures on the type, size, and proportions of the components. Analysis of SCC’s self-compacting capability using two types of lightweight coarse aggregate with different densities showed that as the density of the lightweight aggregate [36] decreased, flowability improved, but the ability to resist segregation decreased. This creates a task to establish the effect of formulation factors on concrete’s technological characteristics, rheological indicators, and structural changes.

Of particular interest are lightweight structural concretes with reduced density (less than 1600 kg/m^3^). As demonstrated by [21], high strength values are achievable on hollow lightweight aggregates such as microspheres. Unlike artificial or natural lightweight fillers, hollow microspheres have a regular particle shape, a different surface morphology, and a significantly smaller size. Lightweight concretes with hollow microspheres can have high strength at a low average density, but increasing the share of the lightweight fraction (up to 50% by volume) increases the risk of segregation of highly fluid concrete mixtures. Thus, the development of structural lightweight concrete with self-compacting ability is of scientific and practical interest.

Establishing the interdependence of the dense and hollow components of concrete with its mobility and homogeneity is a valid task. Establishing the distinctive signs of self-compacting cement systems on hollow aggregates is important since the share of dense components, which contribute greatly to flowing under gravity, decreases. This research aimed to establish the technological and rheological characteristics of concrete mixtures on hollow microspheres compared with heavy fine-grained concretes.

## 2. Materials and Methods

### 2.1. Materials

#### 2.1.1. Components for Concrete

This work is a continuation of research on developing structural LWSCC based on previously obtained results [30]. To establish the influence of formulation factors on cement dispersive systems with increased fluidity on lightweight aggregates, lightweight concretes of different densities with a variable lightweight filler—hollow microspheres—were used as the research object [37,38,39]. The compositions of the study concrete mixtures contained the following components: Portland cement CEM I 42.5 N (PC), ForeSphere aluminosilicate microspheres (MSs), complex silica additive FremSilica-2 (SA), fractional sand (S_f_), quartz powder (S_p_), hyperplasticizer Melflux 2651F (Pl), and water (W).

The binding substance was an additive-free Portland cement, CEM I 42.5 N, produced by JSC Maltsevsky Portland cement and compliant with the chemical and mineralogical composition presented in Table 1; the main properties are presented in Table 2.

The complex silica additive FremSilica 2 is comprised of fine spherical particles of amorphous silica with a specific surface area of 20 m^2^/g, compliant with TU BY 190669631.001-2011, with an average size of about 0.1 μm [40].

**Figure 1:**
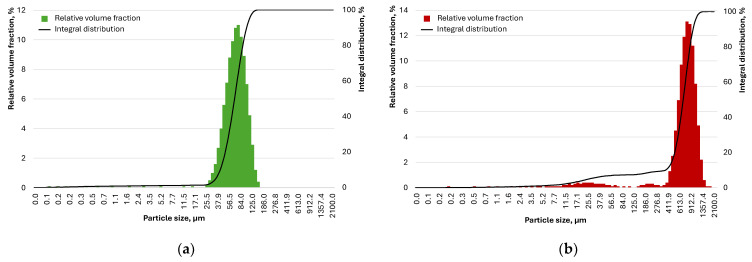
The particle size distribution of microspheres (**a**) and fractionated quartz sand (**b**).

Hollow aluminosilicate ForeSphere microspheres with particle diameters of 10–200 μm (the particle size distribution is shown in Figure 1a) were used as lightweight aggregates to reduce the average density, and the primary characteristics are presented in Table 3.

Fractionated quartz sand was used as a dense filler. The particle size distribution of the sand is shown in Figure 1b. Quartz powder is fine sand obtained by milling fractionated quartz sand in a ball mill to a specific surface area of 720 m^2^/kg.

To manage the technological properties of study concrete mixtures, hyperplasticizer Melflux 2651F was used, which is a second-generation polycarboxylate ether that does not affect cement setting [42].

#### 2.1.2. Composition of Concrete

The ratio of dry components in the compositions was varied depending on the target density by changing the content of hollow microspheres. The compositions of the studied concretes are presented in Table 4.

To assess the influence of formulation factors on the technological and rheological properties of concrete mixtures with different hollow microsphere contents, the amount of water and the plasticizer concentration were kept constant at W/C = 0.5 and *C*_Pl_ = 1.4%. Changing the ratio of fine and fractionated parts of quartz sand allowed the determination of the contribution of components with identical mineral composition to the distribution of water in concretes of different densities. The compositions of the studied concrete mixtures are presented in Table 5.

### 2.2. Methods

#### 2.2.1. Mobility

**Figure 2:**
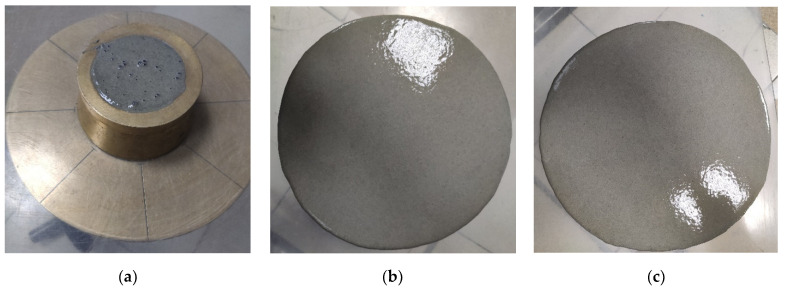
Methodology for determining the mobility of concrete mixtures with hollow microspheres: filling the cone (**a**); spreading diameter after removing the cone (before shaking) (**b**); spreading diameter after 30 shakings (**c**).

The mobility of the concrete mixtures was determined, according to the method in paragraph 1.3 [43], on a shaking table by the spread diameter from a truncated cone with dimensions *D* × *d* × *h* of 101.6 × 69.9 × 50.8 mm [44] before and after 30 shakings (Figure 2).

#### 2.2.2. Rheology

**Figure 3:**
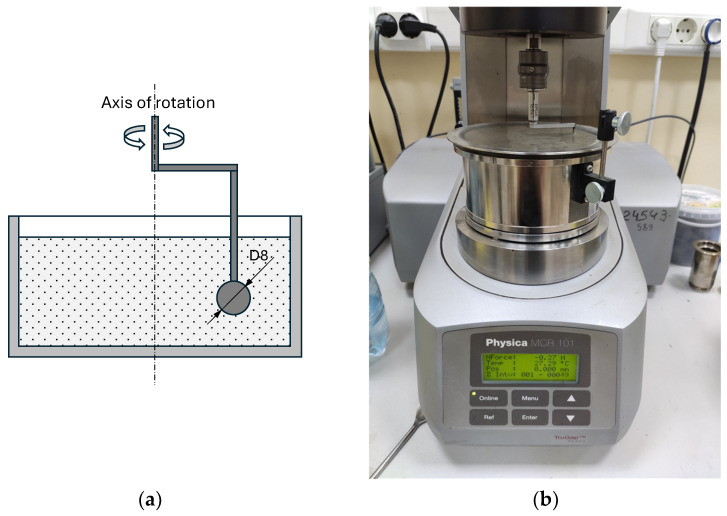
Determination of the viscosity and shear stress of concrete mixture with hollow microspheres by the MCR 101 rotational rheometer: measurement scheme (**a**) and visual appearance (**b**).

The rheological properties were determined using the viscosity and shear stress measurements of the concrete mixtures with an MCR 101 rotational rheometer (Figure 3) during the orbital movement of the measuring system (ball diameter 8 mm) immersed in the concrete mixtures, with linear increments in the shear rate to 1 s^−1^ for 60 s.

Rheological studies were carried out on mixtures immediately after preparation without pause or pre-share. This allowed the results to be correlated with the mobility tests.

#### 2.2.3. Homogeneity

**Figure 4:**
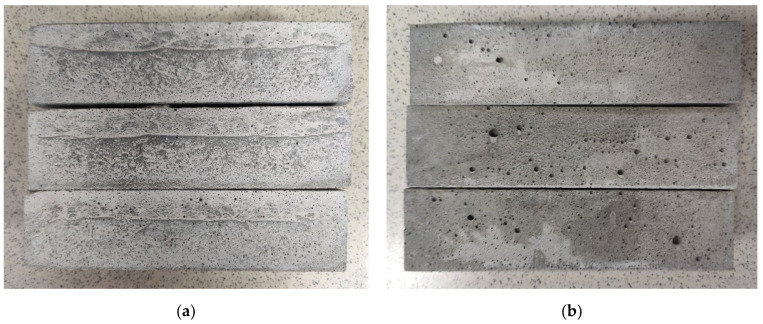
Appearance of lightweight concrete samples with pronounced signs of delamination (**a**) and without them (**b**).

Before determining the mechanical properties, the homogeneity of the obtained samples was qualitatively evaluated. Segregation signs were recorded in cases of the visible separation of a sample of 40 × 40 × 160 mm into two layers of different densities (Figure 4); otherwise, the samples were considered homogeneous.

Homogeneity was evaluated in samples of three series:

- Series 1—samples molded immediately after concrete mixture preparation;
- Series 2—samples molded after testing the mobility of the concrete mixture;
- Series 3—samples molded after determining the shear stress and viscosity of the concrete mixture.

#### 2.2.4. Strength

The estimation of the recipe factors’ influence on the physico-mechanical properties of concrete was carried out on standard prism samples of 40 × 40 × 160 mm without compaction or vibration.

**Figure 5:**
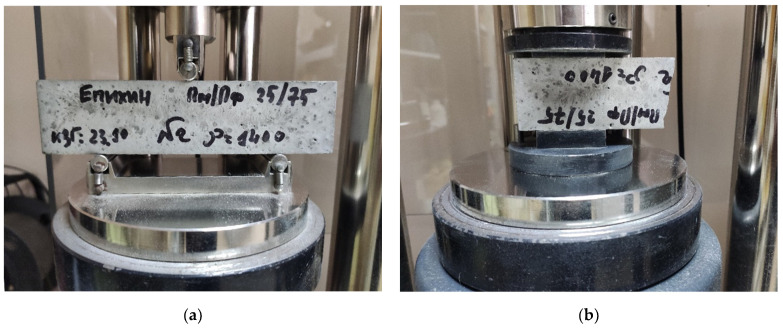
Scheme of testing samples to determine flexural strength (**a**) and compressive strength (**b**).

Flexural and compressive strengths were determined after 28 days of curing in a normal curing chamber using the Advantest 9 servo-hydraulic system (Figure 5), according to standard methods [45].

#### 2.2.5. Statistics

Tests were performed using enough samples to ensure the statistical relevance of the obtained results, as follows: for the spread diameter—4 measurements (standard deviation 1.0…4.9%); for the average density—3 samples (standard deviation 0.1…2.7%); for the flexural strength—3 samples (standard deviation 0.8…5.4%); and for the compressive strength—6 samples (standard deviation 0.5…4.7%).

## 3. Results and Discussion

### 3.1. Technological Properties

A widely accepted criterion for the workability of mixtures based on mineral binders is the spread diameter from a truncated cone. Upon release from the cone under the influence of gravity or additional agitation (shaking), the mixture tends to flow. The intensity of this flow is expressed through a geometric indicator of the formed spread. The presence of agitation during the test creates conditions for forced compaction, for instance, vibration, which promotes thixotropic liquefaction.

The workability of self-compacting mixtures is also evaluated by spread diameter but without shaking, denoted as *D*_sp,1_, which allows for assessing the self-flow capability. Importantly, when evaluating workability by spread diameter both with shaking (*D*_sp,2_) and without it, the key factor is the density of the mixture. The denser the mixture, the more intense its flow under gravity, all other conditions being equal.

**Figure 6:**
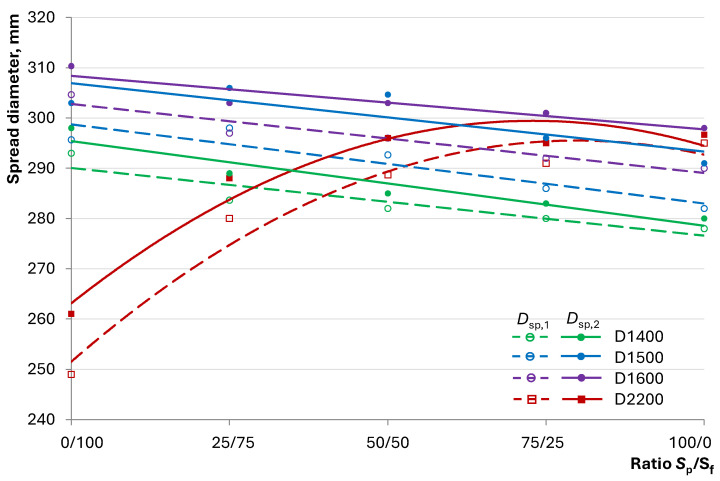
Dependence of the concrete mixture spread diameter on the S_p_/S_f_ ratio.

The compositions of the investigated lightweight concrete mixtures included quartz sand of various finenesses (fractionated and milled), with the total amount varying depending on the target density of the concrete, i.e., the content of hollow microspheres. Meanwhile, the consumption of other dry components of the mixture remained constant, with W/C = 0.5 and *C*_Pl_ = 1.4%. Varying the ratio of fractionated to milled sand allowed for the assessment of the role of the inert component’s fineness in the investigated lightweight concretes. The comparison of the resulting lightweight concrete mixtures was performed with the composition of heavy fine-grained concrete using the same components but without hollow microspheres. The results of the workability investigation for different densities and varying ratios of S_p_/S_f_ are presented in Figure 6.

Figure 6 shows the graphs of the change in the spread diameter of the mixtures before and after shaking obtained for concrete compositions with different densities and varied ratios of quartz powder to fractionated sand. The graphs clearly present distinct patterns of the workability change in heavy (D2200) and lightweight (D1400...D1600) concrete compositions.

The dependence of the spread diameter on the S_p_/S_f_ ratio for heavy concrete is described by an extreme dependency. As the proportion of quartz powder increases, the spread diameter of the mixture increases, but the intensity of this increase diminishes, leading to a decrease in the influence of this factor. This pattern demonstrates the role of quartz powder in ensuring the workability of fine-grained heavy concrete mixtures. According to the basic principle of flow in such systems, their intensity depends on two mutually opposing forces: gravity and friction. The graph shows that increasing S_p_/S_f_, which involves adding more of the component with a larger specific surface area, leads to an increase in *D*_sp,1_ and *D*_sp,2_. This is explained by the greater water-retaining capacity of milled sand compared with that of fractionated sand. On the other hand, the quartz powder in the mixture forms part of the cement–mineral paste that coats the aggregate. Increasing its volume leads to larger distances between the sand grains, thereby increasing the cement–mineral matrix layer, which reduces friction between the particles of fractionated sand. This change is clearly observed when S_p_/S_f_ < 50/50. Further increasing the quartz powder content affects the spread diameter less significantly due to the limitations described above. An excess of the fine component, represented by quartz powder, fails to provide similar positive effects due to increased water adsorption on its surface. As a result, the system experiences a water deficit, expressed by a reduced thickness of the water layer, increasing friction not only between the aggregate grains but also among the cement–mineral matrix particles.

The graphs showing the changes in workability of lightweight concrete mixtures with varying S_p_/S_f_ values indicate that increasing the quartz powder proportion linearly decreases workability. It is evident that denser concrete mixtures have larger spread diameters, which is explained by the gravity’s influence related to the density of the material overall and the filler in particular. Naturally, the higher the hollow microsphere content (lower concrete density), the less workable the mixture.

In contrast to heavy concrete, lightweight concrete includes a lightweight filler with high fineness—hollow microspheres. Consequently, in the absence of quartz powder, the spread diameter of lightweight concrete mixtures is significantly higher than that of heavy concrete, regardless of the density. This suggests that friction forces in such systems are less detrimental. This can be attributed to, on one hand, the spherical shape of the lightweight filler, and on the other hand, the increased distance between the fractionated quartz sand grains resulting from the replacement of dense components with lightweight ones to achieve the required concrete density, reducing the influence of the quartz powder.

It is noteworthy that the patterns of spread diameter change before and after shaking are identical for all compositions. However, the distinction between the *D*_sp,2_ and *D*_sp,1_ graphs for heavy and lightweight concrete is different. The difference between the spread diameter values before and after shaking demonstrates the concrete mixture’s self-compacting ability. This ability can be assessed using a relative indicator—the self-compacting coefficient, calculated by the formula:

*k*_sc_ = *D*_sp,2_/*D*_sp,1_, (1)

where *D*_sp,1_ is the spread diameter of the concrete mixture without shaking and *D*_sp,2_ is the spread diameter after shaking.

**Figure 7:**
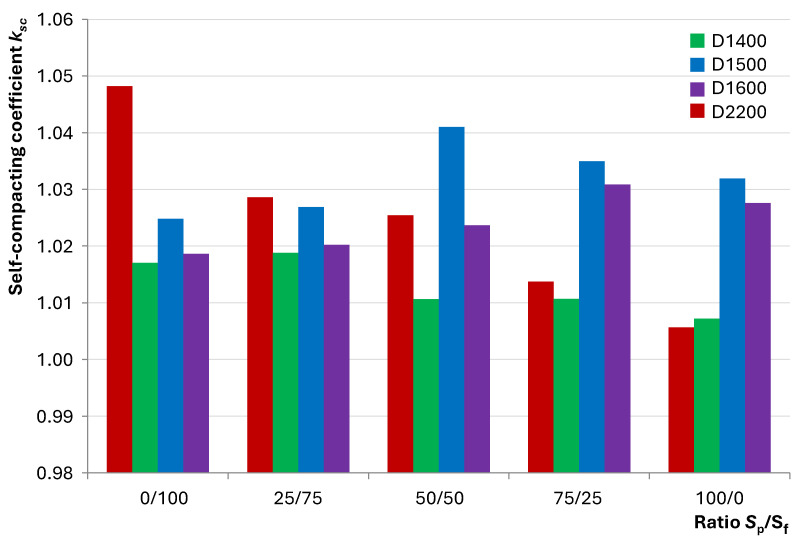
The effect of the S_p_/S_f_ ratio on the self-compacting ability of the studied concrete mixtures.

When *k*_sc_ → 1.0, indicating less of a difference between *D*_sp,2_ and *D*_sp,1_, the mixture’s self-compacting ability is higher, as external mechanical impacts have a less significant influence on workability. Figure 7 shows the dependence of the self-compacting coefficient on S_p_/S_f_, illustrating the described difference.

Figure 7 illustrates that the self-compacting coefficients of heavy concrete mixtures decrease with increasing S_p_/S_f_ ratios, which is explained by reduced frictional resistance, resulting in a less impeded flow. For lightweight concrete mixtures, a different trend is observed. Compositions of varying density exhibit extrema at S_p_/S_f_ ratios of 75/25, 50/50, and 25/75 for D1600, D1500, and D1400, respectively. This shows the variable impact of milled quartz powder in compositions with hollow fillers on the self-compacting coefficient. As the density decreases, the positive effect of the excessive fine fraction outweighs the negative impact, which can be explained by the combined lower proportion of quartz components (Table 4). Therefore, significant differences in the workability of concrete mixtures with dense and hollow fillers, as well as the influence of quartz sand of varying fineness, are established.

### 3.2. Rheological Properties

**Figure 8:**
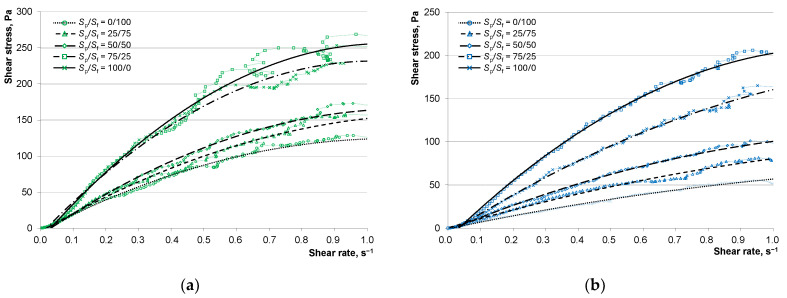

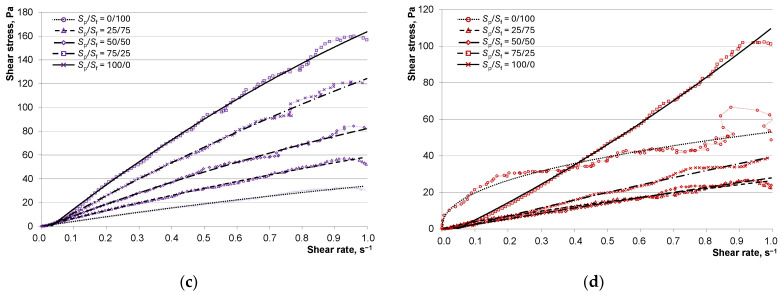
Dependence of shear stress on the shear rate of the concrete mixtures: (**a**) D1400, (**b**) D1500, (**c**) D1600, (**d**) D2200.

To evaluate the changes in rheological characteristics of the investigated concrete mixtures that explain the obtained results, rheological curves were plotted (Figure 8).

A comparative analysis of the rheological indicators was carried out using the Ostwald–de Waele equation [30]:

τ = *k*γ*^n^*, (2)

where τ is the shear stress, γ is the shear rate, *k* is the consistency indicator, and *n* is the indicator of the flow type (*n* < 1 for pseudoplastic flow; *n* > 1 for dilatant flow).

Table 6 presents the parameters for the Ostwald–de Waele equations for each composition, describing the obtained rheological curves.

An analysis of the obtained coefficients for the Ostwald–de Waele equation indicates that the D1400 composition exhibits a dilatant flow behavior (*n* > 1), regardless of the S_p_/S_f_ ratio. This suggests that the structure of such a mixture represents a tightly packed dispersed system in which the liquid phase distributed on the surface of solid particles acts as a lubricant, reducing friction. As the shear rate increases, the packing density decreases, and there is insufficient lubricant for sliding, which is reflected by a more intense increase in shear stress than in shear rate.

For compositions with average densities of 1500 kg/m³ and 1600 kg/m³, the dilatant behavior of the concrete mixture flow changes from pseudoplastic at S_p_/S_f_ = 25/75. At this ratio of quartz powder to fractionated sand, the structure represents dispersed systems in which solid particles have enough cohesion to flow as aggregates. Water may be unevenly distributed on the particle surface, achieving flow in layers, noticeable in the graphs of Figure 7 by a smaller increase in shear stress than in shear rate.

Note that the consistency coefficient *k* for compositions D1500 and D1600 increases with the S_p_/S_f_ ratio. This coefficient similarly depends on the lightweight fraction content; the higher the content of hollow microspheres (lower density), the higher the *k* coefficient, indicating a thicker concrete mixture. This explains the results in Figure 5, showing the decreased workability of mixtures with hollow fillers.

The Ostwald–de Waele equation coefficients for heavy concrete compositions D2200 correspond with the results of the concrete mixture spread diameter studies. Table 6 indicates that varying the S_p_/S_f_ ratio allows for controlling the flow behavior of the mixture, as seen in the transition of the *n* coefficient from values less than 1 to values greater than 1. This, as with lightweight concrete mixtures, indicates a change in the mixture’s structure and, consequently, its flow characteristics. The S_p_/S_f_ ranges 50/50...75/25 correspond to the *k* coefficient extremum, suggesting a reduction in workability with excessive quartz powder, as illustrated above.

**Figure 9:**
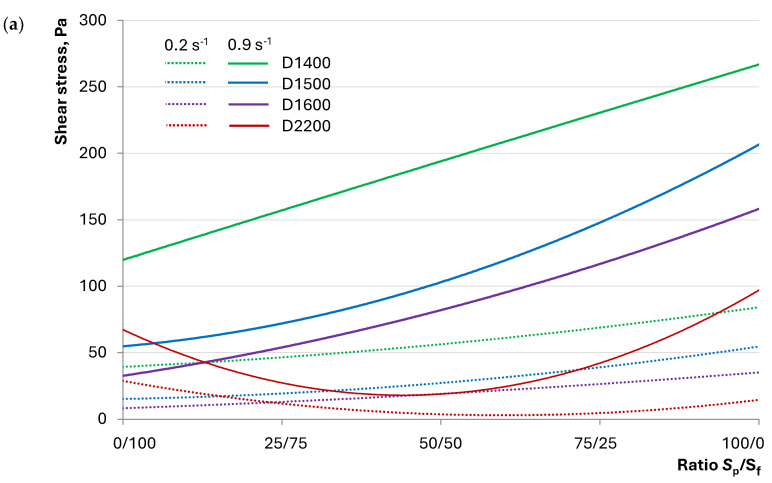

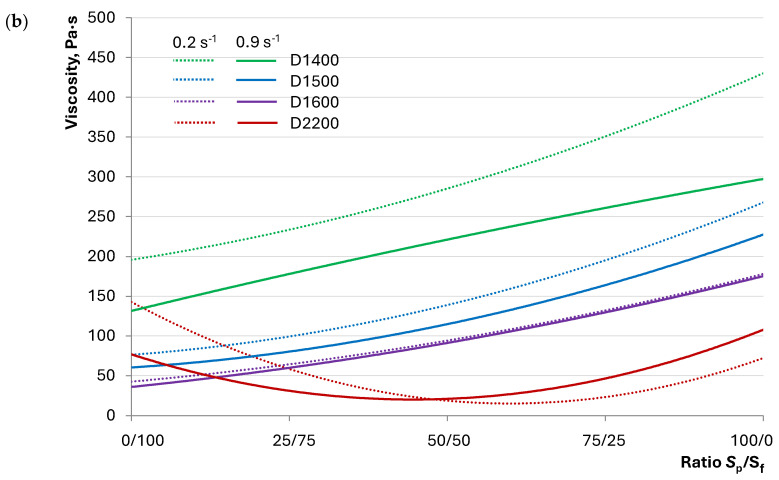
The effect of the S_p_/S_f_—ratio on (**a**) shear stress and (**b**) viscosity of studied concrete mixtures with different shear rates.

Figure 7 shows that compositions with dense fillers differ from those with hollow fillers regarding the S_p_/S_f_ ratio effect. Specifically, the shear stress of lightweight concrete compositions increases with more quartz powder content, while for heavy concrete compositions, it varies nonlinearly. This is evident when comparing shear stress and viscosity graphs at identical shear rates. Besides, viscosity reflects the resistance to flow, i.e., friction forces in the system. Figure 9 shows the rheological properties of heavy and lightweight concretes at shear rates of 0.2 and 0.9 s⁻¹.

The graphs indicate that increasing quartz powder content consistently increases shear stress and viscosity for all lightweight concrete compositions. Simultaneously, more hollow microspheres proportionally increase shear stress and viscosity, as seen in the progressively higher positioning of the D1600, D1500, and D1400 curves.

For heavy concrete compositions, the change in shear stress and viscosity with the S_p_/S_f_ ratio follows a curve with a marked extremum. The transition zone on the graphs, where the trend shifts from decreasing to increasing, lies within the S_p_/S_f_ range of 25/75...75/25, aligning with the *k* and *n* coefficients’ extrema in Table 6. The graphs also demonstrate the structural change in the compositions when varying the S_p_/S_f_ ratio, further supported by the adjustability of the concrete mixture’s behavior.

Thus, these findings underscore significant distinctions in the workability and rheological behavior of concrete mixtures based on dense and hollow fillers, along with the impact of quartz sand fineness.

### 3.3. Homogeneity/Segregation

In the development of SCCs, in addition to high workability, their homogeneity is crucial for practical applications. The tendency of the mixture to segregate, which was used in this study to evaluate homogeneity, was analyzed through the qualitative analysis of the external faces and cross-sections of samples after flexural testing.

The analysis was conducted on samples from three different series, which varied in terms of additional impact on the concrete mixture before placing it into molds and fabricating the samples. This approach allowed for the assessment of the concrete mixture’s behavior after mechanical impact, indirectly simulating external vibrations. The qualitative analysis results are summarized in Table 7.

The qualitative assessment of the macrostructure of lightweight concrete, derived from different density compositions when varying S_p_/S_f_, shows that each composition has a threshold for homogeneity. Compositions with the lowest density, D1400, can be described as homogeneous at any ratio of quartz powder to fractionated sand (Series 1 and 2). However, Series 3 exhibits local inhomogeneities, and at S_p_/S_f_ = 0/100, clear segregation is observed. This behavior may be related to the distribution of water in the system, where the specific surface area of the dry components plays a significant role. In systems with a larger total surface area, the homogeneity is higher, and segregation occurs under more significant external impacts. This is confirmed in compositions D1500 and D1600. However, it should be noted that clear signs of segregation in Series 3 compositions are also observed at S_p_/S_f_ = 25/75, i.e., with an increase in the fraction of fine material. This effect can be explained by the decrease in the proportion of microspheres in denser compositions, which evidently affects the distribution of water on the surface of solid particles. At the same time, local signs of segregation are noted in the D1600 compositions of Series 1 and 2. For heavy D2200 concrete, the macrostructure is described differently. The “±” symbol in Table 7 marks compositions in which the separation of cement slurry is observed without clear signs of segregation, locally or throughout the volume. It is shown that the separation of cement slurry is absent only at the maximum quartz powder content, S_p_/S_f_ = 100/0. This is explained by the role of quartz powder in water adsorption and its distribution throughout the concrete mixture. A lack of fine particles in the concrete mixture contributes to the formation of excess water in the system and its separation.

These signs of segregation correlate with the analysis of rheological curves presented in Table 6. Systems with a more pronounced dilatant flow character are described by greater resistance to segregation.

Therefore, reducing density, i.e., increasing the proportion of hollow microspheres, along with increasing the proportion of quartz powder, can be noted as a factor that enhances the homogeneity of the concrete mixture. However, maintaining the homogeneity of such systems is limited by the intensity of external impacts.

### 3.4. Physical and Mechanical Properties

**Figure 10:**
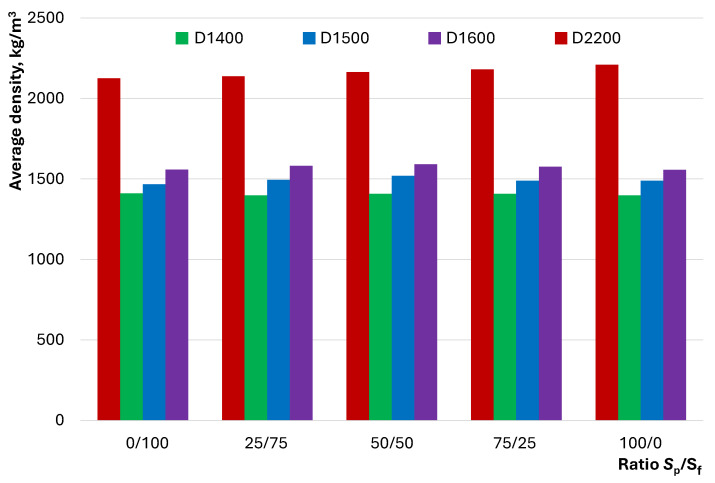
The effect of the S_p_/S_f_ ratio on the average density of the studied concrete.

An important physical parameter of concrete that characterizes its structure is its average density. The effect of the S_p_/S_f_ ratio on the average density of lightweight D1400, D1500, and D1600 concretes compared with heavy D2200 concrete is shown in Figure 10.

Variations in the ratio of quartz powder to fractionated sand for each of the considered compositions lead to slight changes in average density. Figure 10 shows that the effect of S_p_/S_f_ is statistically insignificant; it varies within the range of 2160 ± 35 kg/m³ for heavy concretes and 1405 ± 6 kg/m³, 1495 ± 18 kg/m³, and 1575 ± 15 kg/m³ for lightweight D1400, D1500, and D1600 concretes, respectively. This indicates the insignificance of the dispersion of quartz sand for the investigated compositions with the selected W/C ratio and the content of the plasticizing additive.

**Figure 11:**
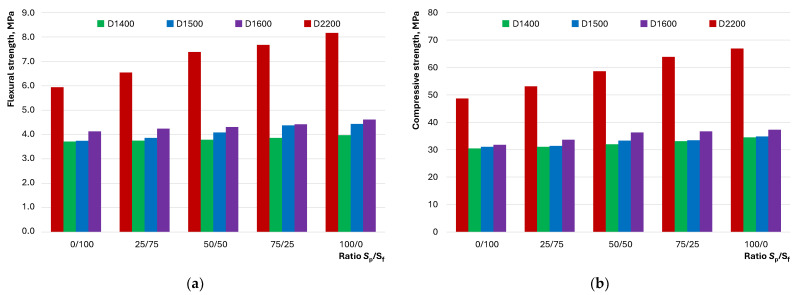
The effect of the S_p_/S_f_ ratio on the (**a**) flexural and (**b**) compressive strengths of the studied concrete.

The workability features of SCC mixtures should not negatively impact the mechanical properties of the concretes to ensure the required structural qualities. The evaluation of the influence of the studied factors, which allow the control of the flowability of lightweight concretes, was carried out by determining the flexural and compressive strengths. Changes in strength indicators based on S_p_/S_f_ are shown in Figure 11.

Predictably, the graphs (Figure 11) show higher values of flexural and compressive strength for heavy concretes (5.9 to 8.2 MPa and 48.6 to 66.9 MPa, respectively) compared with lightweight concretes (3.7 to 4.6 MPa and 30.5 to 37.3 MPa, respectively). It is evident that the influence of the S_p_/S_f_ ratio on the strength of heavy D2200 concrete is more significant. Increasing the proportion of quartz powder from 0/100 to 100/0 leads to a 37…38% increase in both flexural and compressive strength. This indicates that changes in the concrete mixture’s structure positively affect the mechanical properties of heavy concrete. This can be attributed to improvement in the homogeneity of the cement–mineral layer around the quartz sand particles and the water layer on the surface of the solid particles, leading to the formation of an artificial stone structure capable of withstanding greater external mechanical loads. For lightweight concrete compositions D1400…D1600, a similar pattern is observed but with less intensity. It is shown that the increases in flexural and compressive strength are 7…18% and 12…17%, respectively. Moreover, the lower the density of the concrete, the less pronounced the strengthening with an increase in S_p_/S_f_. This effect can be explained by the presence of lightweight aggregate (hollow microspheres). Acting as natural structural defects, the hollow particles lead to earlier failure. Therefore, the positive effect of structure optimization through the introduction of more fine sand particles is reduced by the artificial increase in its defectiveness—the increase in the proportion of hollow microspheres.

**Figure 12:**
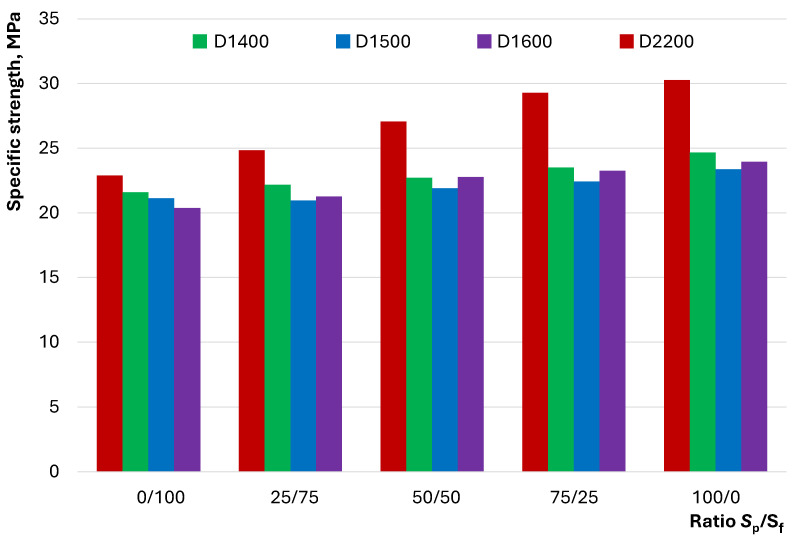
The effect of the S_p_/S_f_ ratio on the specific strength of studied concrete.

A cumulative criterion that allows for the comparison of the structural characteristics of materials with different densities is specific strength. Figure 12 shows changes in specific strength with varying quartz powder and fractionated sand ratios.

Figure 12 shows the differences in the positive impact of changing S_p_/S_f_ on the structural properties of concrete. The specific strength of both heavy and lightweight concretes at S_p_/S_f_ = 0/100 has close values in the range of 20.4 to 22.9 MPa. Increasing the proportion of quartz powder increases the difference between compositions of different densities (D2200 and D1400…D1600). Additionally, it can be seen that lightweight concrete compositions with a D1600 density have lower specific strength than those with D1400, despite having a higher mineral aggregate content. This indicates the ability of such concrete structures to withstand higher loads per unit density. However, none of the lightweight concrete compositions have a specific strength greater than 25 MPa, unlike heavy concrete, which prevents it from being classified as high-strength [21].

Further research on the development of SCC can focus on establishing structural parameters of compositions that allow for achieving both high self-compacting coefficients and high specific strength. Understanding structural features, such as the thickness of the water layer and the cement–mineral layer, and their impact on properties, will enable the development of general principles for producing structural lightweight concretes for monolithic construction.

This research is a continuation of the scientific work [14] on the development of homogeneous and highly mobile structural lightweight concretes. The obtained results complement the research on the influence of formulation factors on the properties of concrete mixtures of structural LWSCC on hollow microspheres [20]. It has been shown that gravitational forces are not a dominant factor for the flow of lightweight concrete mixtures on hollow microspheres, unlike SSC [17]. That is, concrete mixtures with significantly lower aggregate densities can flow more intensively than mixtures with dense aggregates.

## 4. Conclusions

The investigation into the influence of the ratio of dense and hollow aggregates and fine and graded quartz sand on the properties of LWSCC allowed for the formulation of the following conclusions:

- The flow behavior of concrete mixtures with dense and hollow aggregates exhibits distinct characteristics. Lightweight concretes with hollow D1400…D1600 microspheres can exhibit better flowability than heavy D2200 concretes, despite having a lower proportion of dense components—initiators of flow under gravity. An increase in the amount of quartz powder in the composition of lightweight concretes contributes to a decrease in flowability, unlike heavy concretes, in which the pattern follows extremal dependence.
- It was established that the self-compaction coefficient of the mixture of lightweight concrete was comparable to that of heavy concrete. The influence of the ratio of quartz powder to fractionated sand on the self-compaction coefficient of lightweight concrete was described by dependence on an extremum in the range of 50/50...75/25, unlike heavy concrete, for which direct proportionality was observed.
- The rheological curves indicate a dilatant flow behavior for D1400 concrete mixtures regardless of the S_p_/S_f_ ratio. For D1500 and D1600, the dilatant flow behavior shifts to pseudoplastic at S_p_/S_f_ = 25/75. The possibility of controlling the flow type of heavy D2200 concrete by varying the ratio of quartz powder to fractionated sand was demonstrated.
- Differences in the flow behavior of lightweight and heavy concrete mixtures are reflected by the dependence of shear stress and viscosity on S_p_/S_f_. For lightweight concrete compositions, an increase in the content of quartz powder contributes to an increase in shear stress and viscosity, while for heavy concrete, a transition from\descending to ascending dependency is observed in the range of S_p_/S_f_ = 25/75…75/25 at different shear rates.
- The studied compositions of lightweight concrete can be described as homogeneous at any S_p_/S_f_ ratio. However, signs of local inhomogeneity or segregation were observed under significant external impacts for D1500 and D1600 compositions and those with minimal quartz powder content. It was shown that concrete mixtures with a pronounced dilatant flow behavior exhibited greater resistance to segregation.
- The ratio of quartz powder to fractionated sand has a statistically insignificant effect on the average density of the studied concretes. However, flexural and compressive strengths vary significantly with this factor in heavy concretes (up to 38%) compared with lightweight concretes (up to 18%). The specific strength of lightweight and heavy concrete compositions at S_p_/S_f_ = 0/100 has close values in the range of 20.4…22.9 MPa, and an increase in the proportion of quartz powder increases the difference between compositions of different densities.

A promising direction for further research in the development of structural LWSCC could be the determination of structural parameters of compositions, allowing for the production of concretes with both a high self-compaction coefficient and a high specific strength. Structural features such as the thickness of the water layer and the thickness of the cement–mineral layer should be established for their influence on properties, as should the main principles for producing structural lightweight concretes for use in monolithic construction.

## Figures and Tables

**Table 1:** Chemical and mineralogical composition of clinker.

Oxides							Minerals			
CaO	SiO_2_	Al_2_O_3_	Fe_2_O_3_	MgO	SO_3_	Na_2_O	C_3_S	C_2_S	C_3_A	C_4_AF
66.0	21.2	5.1	4.1	0.75	0.56	0.58	68.2	8.2	6.4	12.6

**Table 2:** The main properties of the Portland cement.

No	Property	Value
1	Compressive strength (age is 2 days), MPa	22.9
2	Compressive strength (age is 28 days), MPa	52.6
3	Initial/finish setting time, min	175/300
4	Specific surface area, m^2^/kg	408
5	Normal density of cement dough, %	24.3

**Table 3:** Properties of hollow aluminosilicate ForeSphere microspheres [41].

No	Property	Value
1	Bulk density, kg/m^3^	320…370
2	True density, kg/m^3^	580…690
3	Average particle size, μm	65…70
4	The thickness of the walls of the microsphere, μm	2…10
5	Wall material density, kg/m^3^	2500
6	Compressive strength, MPa	15.0–28.0
7	Mohs scale hardness	5–6

**Table 4:** Ratio of components of the studied mixtures.

No	Composition	Volume Content, %				
		PC	SA	S_p_ + S_f_	MS	W + Pl
1	D1400	20.0	3.1	8.8	46.4	21.7
2	D1500			13.4	41.7	
3	D1600			18.2	37.0	
4	D2200			55.2	0.0	

**Table 5:** The volume content of quartz powder and fractional sand at different values of S_p_/S_f_—the ratio for different compositions of concrete.

No	Composition	Component	Volume Content, %				
			0/100	25/75	50/50	75/25	100/0
1	D1400	S_p_	0.0	2.2	4.4	6.6	8.8
		S_f_	8.8	6.6	4.4	2.2	0.0
2	D1500	S_p_	0.0	3.35	6.7	10.05	13.4
		S_f_	13.4	10.05	6.7	3.35	0.0
3	D1600	S_p_	0.0	4.55	9.1	13.65	18.2
		S_f_	18.2	13.65	9.1	4.55	0.0
4	D2200	S_p_	0.0	13.8	27.6	41.4	55.2
		S_f_	55.2	41.4	27.6	13.8	0.0

**Table 6:** The volume content of quartz powder and fractional sand at different S_p_/S_f_ ratios in different compositions of concrete.

No	Composition	Coefficients	S_p_/S_f_				
			0/100	25/75	50/50	75/25	100/0
1	D1400	*k*	174.0	207.8	237.7	359.7	396.4
		*n*	1.04	1.09	1.16	1.19	1.22
2	D1500	*k*	65.4	97.8	127.6	204.8	283.8
		*n*	0.99	1.04	1.07	1.19	1.22
3	D1600	*k*	36.4	63.8	88.2	151.0	212.9
		*n*	0.95	1.00	1.01	1.21	1.28
4	D2200	*k*	52.0	26.7	24.4	45.1	121.2
		*n*	0.42	0.83	0.87	1.22	1.42

**Table 7:** Signs of segregation of studied concrete compositions with varying S_p_/S_f_ ^1^.

No	Composition	Series	S_p_/S_f_				
			0/100	25/75	50/50	75/25	100/0
1	D1400	Series 1	No	No	No	No	No
2		Series 2	No	No	No	No	No
3		Series 3	+	±	±	±	±
4	D1500	Series 1	No	No	No	No	No
5		Series 2	No	No	No	No	No
6		Series 3	+	+	±	±	±
7	D1600	Series 1	±	No	No	No	No
8		Series 2	+	±	No	No	No
9		Series 3	+	+	+	±	±
10	D2200	Series 1	±	±	±	±	No
11		Series 2	±	±	±	±	No
12		Series 3	±	±	±	±	±

^1^ “+” shows compositions with clear signs of segregation; “±” shows compositions with local inhomogeneities or the separation of cement slurry.

## Data Availability

The original contributions presented in the study are included in the article, further inquiries can be directed to the corresponding author.
